# On the Virtualization of Audio Transducers

**DOI:** 10.3390/s23115258

**Published:** 2023-06-01

**Authors:** Riccardo Giampiccolo, Alberto Bernardini, Oliviero Massi, Augusto Sarti

**Affiliations:** Dipartimento di Elettronica, Informazione e Bioingegneria (DEIB), Politecnico di Milano, Piazza L. Da Vinci 32, 20133 Milano, Italy; riccardo.giampiccolo@polimi.it (R.G.); oliviero.massi@polimi.it (O.M.); augusto.sarti@polimi.it (A.S.)

**Keywords:** circuital inversion, sensor virtualization, actuator virtualization, audio transducers, digital signal processing

## Abstract

In audio transduction applications, virtualization can be defined as the task of digitally altering the acoustic behavior of an audio sensor or actuator with the aim of mimicking that of a target transducer. Recently, a digital signal preprocessing method for the virtualization of loudspeakers based on inverse equivalent circuit modeling has been proposed. The method applies Leuciuc’s inversion theorem to obtain the inverse circuital model of the physical actuator, which is then exploited to impose a target behavior through the so called Direct–Inverse–Direct Chain. The inverse model is designed by properly augmenting the direct model with a theoretical two-port circuit element called nullor. Drawing on this promising results, in this manuscript, we aim at describing the virtualization task in a broader sense, including both actuator and sensor virtualizations. We provide ready-to-use schemes and block diagrams which apply to all the possible combinations of input and output variables. We then analyze and formalize different versions of the Direct–Inverse–Direct Chain describing how the method changes when applied to sensors and actuators. Finally, we provide examples of applications considering the virtualization of a capacitive microphone and a nonlinear compression driver.

## 1. Introduction

Audio transducers are devices that convert electrical signals into acoustic waves or vice versa [1]. In the first case, they are called *audio actuators* (e.g., loudspeakers), while in the second case *audio sensors* (e.g., microphones). The transduction process that characterizes such devices involves different physical domains (such as mechanical, acoustic, electrical, magnetic, etc.), which not only are affected by different nonlinear behaviors but they do interact in a nonlinear fashion. For instance, piezoelectric loudspeakers are impaired by hysteretic phenomena which do increase the Total Harmonic Distortion (THD) [2,3,4], electrodynamic loudspeakers are characterized by a nonlinear force factor and compliance [5], while clipping is the major source of distortion in microphones [6]. Audio transducers are pervasive devices that have become, over the years, of fundamental importance all over the markets. It is thus desirable to come up with solutions that enable a control on the nonlinear behavior of acoustic transducers, and thus on the amount of distortion that they introduce, such that better acoustic performance can be obtained.

Since the rise of the audio industry, different techniques have been proposed for improving the sonic response of audio transducers. Apart from solutions based on a more refined analog design, many techniques do exploit digital audio signal processing for accomplishing such a task. For the case of audio actuators, the simplest solutions make use of filters to equalize the acoustic response over the frequency spectrum [7]. Other solutions, instead, pre-distort the electrical signal with the aim of reducing the impact of nonlinearities [8]; others involve feedback loops for accomplishing linearization and compensation [9,10], while more recent virtual bass enhancement techniques exploit psychoacoustic effects for deceiving the human perception of sound [11,12,13]. On the other side, similar approaches have been proposed for digitally enhancing the performance of audio sensors [14,15].

In this work, we introduce and analyze a novel class of digital signal processing algorithms which we refer to as *virtualization* algorithms. We define virtualization as the task of digitally altering and conditioning the acoustic behavior of an audio transducer with the aim of mimicking the sound of a (virtual) target transducer. Such algorithms are based on general signal processing chains which can be exploited to perform all the traditional tasks envisaged by the algorithms mentioned in the previous paragraph, e.g., linearization and equalization. Recently, loudspeaker virtualization has been tackled by using a digital signal processing approach based on physical modeling [16,17], which exploits the inverse model of the loudspeaker equivalent circuit. The design of the inverse system relies on Leuciuc’s theorem [18], reworded in [16,19], and it is derived by duly adding to the direct circuital system a theoretical two-port element, known in circuit theory as *nullor*. The digital inverse system can then be used to compensate for the behavior of the physical loudspeaker and, hence, impose the behavior of a digital target system. This is achieved by implementing the so called Direct–Inverse–Direct Chain [17] composed of a target direct system, which is a digital filter characterized by the desired transduction behavior to be imposed; the inverse loudspeaker system, which is a digital filter whose response is the inverse of that of the physical transducer; and the (direct) phyisical loudspeaker. While in the approach of [16,17] inversion is digitally attained, other methods to design inverse circuital systems, which rely on analog filters or integrated circuits, such as operational transconductance amplifiers, current conveyors, current differencing buffered amplifiers, etc. [20], have been proposed. However, for the sake of simplicity, in this manuscript, we will only consider inverse design approaches based on digital filters. In this regard, nullors can be efficiently implemented in the discrete-time domain making use of the Wave Digital Filter (WDF) paradigm.

WDF theory was originally introduced by A. Fettweis in the late 1970s for designing stable digital filters through the discretization of linear passive analog filters [21], and was later extended to also efficiently implement active and nonlinear circuits in the discrete-time domain [22,23,24]. In the WDF framework, port voltages and port currents are substituted with linear combinations of *incident* and *reflected waves* introducing a free parameter per port called *port resistance*. In the Wave Digital (WD) domain, circuit elements are modeled in a modular fashion as input–output blocks characterized by scattering relations, while topological interconnections or, more generally, connection networks are described by multi-port junctions characterized by scattering matrices [21,24]. The introduced free parameters can be properly set to eliminate some delay-free-loops (i.e., implicit relations between circuit variables) appearing in the digital structure composed of input–output elements and junctions. Circuits with up to one nonlinear element (described by an explicit mapping) can be digitally implemented in the WD domain in a fully explicit fashion, i.e., without making use of any iterative solver [24], while using stable discretization methods (e.g., Backward Euler, trapezoidal rule, etc.) to approximate time-derivatives. As far as the implementation of nullors is concerned, different techniques have been proposed in the literature of WDFs. In [22], stamps are provided for encompassing nullors into scattering junctions by means of the Modified Nodal Analysis (MNA) formalism. The same result is reached in a more efficient fashion considering a double digraph decomposition of the connection network, as pointed out in [23]. In [25], vector waves are used to derive a vectorial scattering relation that allows to implement a nullor as a two-port input–output element in the WD domain. Moreover, in [16,17], it has already been shown that WDFs are suitable to efficiently emulate direct and inverse models of nonlinear loudspeakers in the discrete-time domain with no need of iterative solvers.

In this paper, we discuss the task of audio transducer virtualization from a general theoretical perspective, by analyzing different scenarios and combinations of input/output signals. Our aim is to provide a *compendium* for the design of inverse circuital models of audio transducers in different vitualization scenarios. In fact, we describe both the case of actuator virtualization and of sensor virtualization, making appropriate adjustments to the employed Direct–Inverse–Direct Chain [17,26]. In doing so, we will consider electrical equivalent models of audio transducers which are derived by exploiting the electro-mechano-acoustical analogy [1,27]. Finally, we present two case studies showing how the proposed methodology can be exploited to alter the acoustic response of different audio devices.

The manuscript is organized as follows. Section 2 provides, first, background knowledge on nullor-based inversion of circuital systems, and, then, introduces ready-to-use schemes and block diagrams for the inversion of electrical equivalent models of audio systems taking into account all possible combinations of input and output variables. Virtualization algorithms that exploit nullor-based inversion of circuits are, instead, presented in Section 3. Examples of application of such algorithms are provided in Section 4 and Section 5, where the virtualization of a capacitive microphone and a nonlinear compression driver are presented. Conclusions are drawn in Section 6.

## 2. Nullor-Based Inversion of Circuital Systems

In this section, we first provide background knowledge on nullors, and, then, we present the four major classes of inversion scenarios, supplementing the overview that is available in the literature which only comprises two cases out of four [18].

### 2.1. Nullors

**Figure 1:**
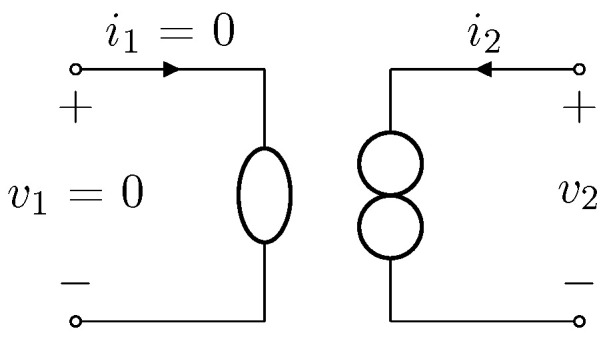
Nullor circuit symbol. The nullator (port 1) is represented with an ellipse, while the norator (port 2) with two circles.

Nullors are theoretical two-port elements composed of other two theoretical one-ports: a *nullator*, which has both port voltage and port current equal to zero, and *norator*, which is characterized by unconstrained port variables [28]. Figure 1 shows the circuital symbol of a nullor, where the nullator (on the left) is represented by means of an ellipse, and the norator (on the right) by means of two circles. The constitutive equation of such a two-port can be thus written as

(1) $$\left[\begin{array}{c} v_{1}\\ i_{1} \end{array}\right]=\left[\begin{array}{cc} 0 & 0\\ 0 & 0 \end{array}\right]\left[\begin{array}{c} v_{2}\\ i_{2} \end{array}\right]\;,$$

where $v_{1}$ and $i_{1}$ are the voltage across and the current through the nullator, whereas $v_{2}$ and $i_{2}$ are the voltage across and the current through the norator. It is worth mentioning that, while nullator and norator do not correspond to physical elements, if properly used, nullors do describe physical devices. In fact, nullors are typically employed in circuit theory to model the ideal behavior of some multi-ports (active and passive), such as Operational Amplifiers (opamp), transistors operating in linear regime, gyrators, transconductance amplifiers, etc. [28,29,30].

Nullor is the fundamental element for carrying out the inversion of circuits according to Leuciuc’s theorem [17,18,19]. Although the main application of the aforementioned circuit inversion method originally was the synchronization of non-autonomous chaotic circuits (i.e., chaotic circuits with exogenous input) in analog secure communication systems [18,31], the method can be generally applied to design the inverse, if this exists, of whatever linear or nonlinear non-autonomous circuit.

In the following subsections, we will reword the original theorem considering the four possibile combinations of input and output signals of the system to be inverted, providing proofs and a complete overview over the method.

### 2.2. Inversion Theorem

**Figure 2:**
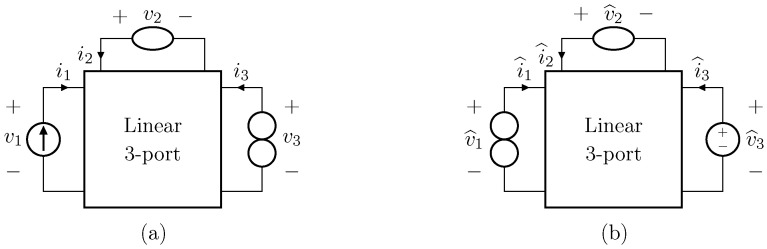
Example of circuital inversion. (**a**) *Direct System*; (**b**) *Inverse System*.

Before presenting the nullor-based inversion theorem, let us consider the two linear-time-invariant (LTI) non-autonomous circuits shown in Figure 2. The input and output of the circuit shown in Figure 2a, which we call *Direct System*, are $i_{1}$ and $v_{3}$, whereas the input and output of the circuit shown in Figure 2b, which we call *Inverse System*, are ${\hat{v}}_{3}$ and ${\hat{i}}_{1}$, respectively. The only difference between the two networks is the position of the three depicted one-port elements, meaning that the remaining part of the circuit is the same for both the two systems. Naming Z as the impedance matrix of the *Direct System*, we can write down the following system of equations

(2) $$\left\{\begin{array}{c} v_{1}=z_{11}i_{1}+z_{12}i_{2}+z_{13}i_{3}\\ v_{2}=z_{21}i_{1}+z_{22}i_{2}+z_{23}i_{3}\\ v_{3}=z_{31}i_{1}+z_{32}i_{2}+z_{33}i_{3} \end{array}\right.,$$

where $z_{11},\ldots ,z_{33}$ are entries of matrix Z, and the time dependence is removed for the sake of clarity. Then, the nullator sets $v_{2}=0$ and $i_{2}=0$, which allow us to derive the transfer function F(s) and the transfer impedance H(s) in the Laplace domain as follows

(3) $$\begin{array}{cc} F(s) & =\frac{I_{3}\left(s\right)}{I_{1}\left(s\right)}=-\frac{z_{21}}{z_{23}}\;, \end{array}$$

(4) $$\begin{array}{cc} H(s) & =\frac{V_{3}\left(s\right)}{I_{1}\left(s\right)}=\frac{z_{23}z_{31}-z_{21}z_{33}}{z_{23}}\;. \end{array}$$

In Equation (2), $v_{x}$ and $i_{x}$ are the voltage and current at port *x* of the network shown in Figure 2a. We can repeat the same procedure for the circuit shown in Figure 2b yielding

(5) $$\left\{\begin{array}{c} {\hat{v}}_{1}=z_{11}{\hat{i}}_{1}+z_{12}{\hat{i}}_{2}+z_{13}{\hat{i}}_{3}\\ {\hat{v}}_{2}=z_{21}{\hat{i}}_{1}+z_{22}{\hat{i}}_{2}+z_{23}{\hat{i}}_{3}\\ {\hat{v}}_{3}=z_{31}{\hat{i}}_{1}+z_{32}{\hat{i}}_{2}+z_{33}{\hat{i}}_{3} \end{array}\right.,$$

and then, by recalling that ${\hat{v}}_{2}=0$ and ${\hat{i}}_{2}=0$ hold true, we can obtain the transfer function $\hat{F}\left(s\right)$ and transfer impedance $\hat{H}\left(s\right)$ in the Laplace domain as follows

(6) $$\begin{array}{cc} \hat{F}\left(s\right) & =\frac{{\hat{I}}_{1}\left(s\right)}{{\hat{I}}_{3}\left(s\right)}=-\frac{z_{23}}{z_{21}}=F^{-1}\left(s\right)\;, \end{array}$$

(7) $$\begin{array}{cc} \hat{H}\left(s\right) & =\frac{{\hat{I}}_{1}\left(s\right)}{{\hat{V}}_{3}\left(s\right)}=\frac{z_{23}}{z_{23}z_{31}-z_{21}z_{33}}=H^{-1}\left(s\right)\;. \end{array}$$

In Equation (5), ${\hat{v}}_{x}$ and ${\hat{i}}_{x}$ are the voltage and current at port *x* of the network shown in Figure 2b. Equation (7) proves the circuit in Figure 2b to have a transfer impedance equal to $H^{-1}\left(s\right)$. We thus conclude that the circuit shown in Figure 2b is the inverse of the circuit shown in Figure 2a. Finally, it is worth pointing out that, in order for this to hold true, the transfer function of the direct system must be minimum phase [18].

Hereafter, we first introduce a reworded version of Leuciuc’s theorem which also generalizes the above approach for the inversion of linear circuits to the case of nonlinear circuits, and then we go through possible inversion scenarios characterized by pairs of input/output variables of different kinds.

**Figure 3:**
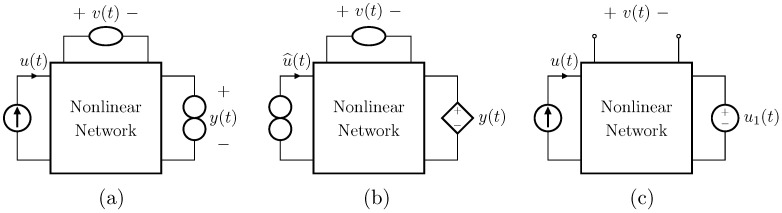
(**a**) *Direct System* containing one nullor; (**b**) *Inverse System*; (**c**) circuit employed for the Proof of Theorem 1.

**Theorem** **1.** *Let us consider a nonlinear non-autonomous circuit containing at least one nullor, as the one shown in Figure 3a. Let us also consider the circuit shown in Figure 3b, where the input generator is replaced by a norator, and the norator by a proper controlled source. If for any input signal u(t) and y(t), where t is the continuous-time variable in seconds, such systems have unique bounded solutions, and if, defined the state vectors of the two systems as x(t) and $\hat{x}\left(t\right)$, the equation $x\left(0\right)=\hat{x}\left(0\right)$ holds true, then the circuit in Figure 3b is the inverse of the circuit in Figure 3a.*

**Proof.**  Let us remove, for the moment, the nullator and the norator from the circuit shown in Figure 3a, and let us replace the norator with a voltage source $u_{1}$. The circuit that we obtain is shown in Figure 3c. By considering voltage *v* as the output, we can describe such a system according to the state-space formalism as follows

(8) $$\left\{\begin{array}{c} \dot{x}=f\left(x,u,u_{1}\right)\;\;\mathrm{with}\;f:R^{n}\times U\times U_{1}\to R^{n}\;\\ v=g\left(x,u,u_{1}\right)\;\;\mathrm{with}\;f:R^{n}\times U\times U_{1}\to R^{n}\; \end{array}\right.$$

where *U* and $U_{1}$ are the sets of input signals *u* and $u_{1}$ that are admissible for the considered application. Note that, for the sake of clarity, here and in the following, we omit the dependence on the continuous-time variable *t*. If we re-introduce the nullator, we constrain voltage *v* to be zero leading the system into an unphysical condition. In order to avoid such an unphysical state, one of the two sources *u* and $u_{1}$ must be substituted with a norator. This will in turn assume voltage and current values such that the system of Equation (8) presents real solutions. It follows that two possible circuits can be derived, namely, the circuits in Figure 3a,b. Given that these two systems are characterized by the same topology, we can write for the circuit shown in Figure 3a

(9) $$\left\{\begin{array}{c} \dot{x}=f\left(x,u,y\right)\\ 0=g(x,u,y) \end{array}\right.,$$

while for the circuit shown in Figure 3b

(10) $$\left\{\begin{array}{c} \dot{\hat{x}}=f\left(\hat{x},\hat{u},y\right)\\ 0=g(\hat{x},\hat{u},y) \end{array}\right..$$

If the two circuits have a well-defined behavior, y=h(x,u) is the only possible solution for equation g(x,u,y)=0 for each $x\in R^{n}$. Moreover, assuming h(x,u) invertible with respect to *u* on the entire state space, we can write $u=h^{-1}\left(x,y\right)$; it follows that $\hat{u}=h^{-1}\left(\hat{x},y\right)$ is the only possible solution for equation $g(\hat{x},\hat{u},y)=0$ for each $\hat{x}\in R^{n}$ [18]. In the light of these considerations, Equation (9) can be rewritten as

(11) $$\left\{\begin{array}{c} \dot{x}=f\left(x,u,h\left(x,u\right)\right)\\ y=h(x,u) \end{array}\right.,$$

and Equation (10) as

(12) $$\left\{\begin{array}{c} \dot{\hat{x}}=f\left(\hat{x},h^{-1}\left(\hat{x},y\right),y\right)\\ \hat{u}=h^{-1}\left(\hat{x},y\right) \end{array}\right..$$

Therefore, if *y* is a bounded solution for Equation (9), and Equation (10) has a bounded solution too, it follows that, for $x\left(0\right)=\hat{x}\left(0\right)$, $\hat{u}=u$ is a solution of Equation (10). □

It is worth pointing out that, although the theorem and proof are referred to the circuits shown in Figure 3, they can be applied to all the four possible inversion scenarios characterized by pairs of input/output variables of different kinds. Table 1 provides an overview over such four cases, associating each type of *Direct System* to its corresponding *Inverse System*, according to input and output variables. In the next subsections, we will address each of the cases one at a time.

#### 2.2.1. Voltage Input Voltage Output (VIVO)

Let us consider Network A of Table 1 where the *Direct System* features both as input and output a voltage signal, namely, $V_{\mathrm{in}}$ and $V_{\mathrm{out}}$. This is one of the original examples taken into account by Leuciuc for deriving the inversion theorem [18], and later employed in [16,17] for deriving the loudspeaker virtualization algorithm. Then, let us consider the case in which a nullor is already present into the *Direct System*. For this particular case, the *Inverse System* is obtained by replacing the input voltage source of the *Direct System* with the norator and the norator with a Voltage-Controlled Voltage Source (VCVS) driven by the output voltage of the *Direct System*. The inverse of Network A is Network B of Table 1.

**Figure 4:**
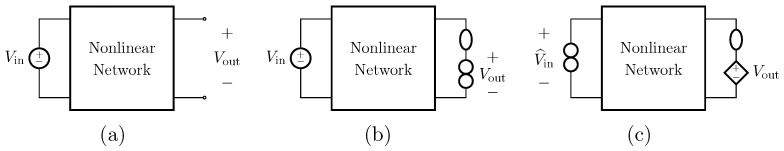
Augmenting a circuit with a series connection of nullator and norator. (**a**) Circuit presenting no nullor; (**b**) circuit with nullor equivalent to the circuit in (**a**); (**c**) inverse of the circuit in (**b**) and, in turn, of the circuit in (**a**).

In the case in which no nullors are present into the *Direct System* (see Figure 4a), this can be augmented with the series connection of a nullator and a norator, as shown in Figure 4b. In fact, this adjunct does not modify the behavior of the circuit since the series of a nullator and a norator is equivalent to an open circuit [30]. Such a series connection must be inserted between the very same nodes where the output voltage is taken [18]. Then, the *Inverse System* is obtained following the same procedure described for the case of circuits already containing nullors. Finally, Figure 4c shows the inverse of the circuit in Figure 4a.

#### 2.2.2. Voltage Input Current Output (VICO)

Let us consider Network C of Table 1 where the *Direct System* features as input a voltage signal and as output a current signal, namely, $V_{\mathrm{in}}$ and $I_{\mathrm{out}}$. Even this scenario is part of the original examples taken into account by Leuciuc in [18]. Let us first consider the case in which a nullor is already present into the *Direct System*. For this particular case, the *Inverse System* is obtained by replacing the input voltage source of the *Direct System* with the norator and the norator with a Current-Controlled Current Source (CCCS) driven by the output current of the *Direct System*. The inverse of Network C is Network D of Table 1.

**Figure 5:**
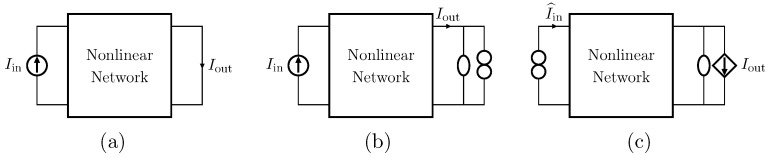
Augmenting a circuit with a parallel connection of nullator and norator. (**a**) Circuit presenting no nullor; (**b**) circuit with nullor equivalent to the circuit in (**a**); (**c**) inverse of the circuit in (**b**) and, in turn, of the circuit in (**a**).

In the case in which no nullors are present into the *Direct System*, this can be augmented with the parallel connection of a nullator and a norator. In fact, this adjunct does not modify the behavior of the circuit given that a nullator and a norator in parallel are equivalent to a short circuit [30]. Such a parallel connection must be inserted in series with the very same branch through which the output current flows [18]. The result will be a circuit similar to the one shown in Figure 5b but with a voltage input, while the *Inverse System* will be similar to the one shown in Figure 5c but considering the voltage across the norator instead of the current through it.

#### 2.2.3. Current Input Voltage Output (CIVO)

Let us consider Network E of Table 1 where the *Direct System* features as input a current signal and as output a voltage signal, namely, $I_{\mathrm{in}}$ and $V_{\mathrm{out}}$. Let us first consider the case in which a nullor is already present into the *Direct System*. For this particular case, the *Inverse System* is obtained by replacing the input current source of the *Direct System* with the norator and the norator with a VCVS driven by the output voltage of the *Direct System*. The inverse of Network E is Network F of Table 1.

In the case in which no nullors are present into the *Direct System*, this can be augmented with the series connection of a nullator and a norator. As for the VIVO case, such a series connection must be inserted between the very same nodes where the output voltage is taken. The result will be a circuit similar to the one shown in Figure 4b but with a current input, while the inverse system will be similar to the one shown in Figure 4c, but considering the current flowing through the norator instead of the voltage across it.

#### 2.2.4. Current Input Current Output (CICO)

Let us consider Network G of Table 1 where the *Direct System* features both as input and output a current signal, namely, $I_{\mathrm{in}}$ and $I_{\mathrm{out}}$. Let us first consider the case in which a nullor is already present into the *Direct System*. For this particular case, the *Inverse System* is obtained by replacing the input current source of the *Direct System* with the norator and the norator with a CCCS driven by the output current of the *Direct System*. The inverse of Network G is Network H of Table 1.

In the case in which no nullors are present into the *Direct System* (see Figure 5a), this can be augmented with the parallel connection of a nullator and a norator. As for the VICO case, such a parallel connection must be inserted in series with the very same branch through which the output current flows [18], as shown in Figure 5b. Then, the *Inverse System* is obtained following the same procedure described for the case of circuits already containing nullors. Finally, Figure 5c shows the inverse of the circuit in Figure 5a.

### 2.3. Adjoint Networks

In this subsection we make some considerations on homogeneous inversion scenarios, where the input and the output variables do have the same units of measurement, i.e., VIVO and CICO scenarios. In these cases, *adjoint networks* can be considered for transforming a voltage-voltage transfer function into a current-current transfer function and vice versa. In fact, the nature of the transfer function will have implications as far as implementation is concerned. For instance, voltages and currents might be characterized by different orders of magnitude and, thus, working with the former may be more convenient than working with the latter or vice versa, especially when the *Inverse System* is implemented in the digital domain. Entering more in detail, two *N*-port networks α and β are called *adjoint* if the following equation holds true [32]

(13) $$\sum_{n=1}^{N}\left(v_{\alpha ,n}i_{\beta ,n}-i_{\alpha ,n}v_{\beta ,n}\right)=0\;,$$

where $v_{\alpha ,1},\ldots ,v_{\alpha ,N}$ and $i_{\alpha ,1},\ldots ,i_{\alpha ,N}$ are the port voltages and port currents of network α, whereas $v_{\beta ,1},\ldots ,v_{\beta ,N}$ and $i_{\beta ,1},\ldots ,i_{\beta ,N}$ are the port voltages and port currents of network β, respectively. According to Equation (13), for example, we can expect the adjoint of an ideal voltage amplifier to maintain the same topology but act as an ideal current amplifier.

The procedure for deriving the adjoint of a given circuit, whose input is a voltage signal, can be summarized as follows:

- Passive elements are kept without any changes.
- Nullators are replaced with norators, while norators with nullators.
- The input voltage is replaced with a short circuit (i.e., a current sink). The output of the adjoint circuit will be then the current flowing through such a short, where the positive direction follows the element convention, i.e., from the positive to the negative terminal.
- A current source is connected to the output port. This will be the input of the adjoint circuit. In this case, the direction of the current follows the source convention, i.e., from the negative to the positive terminal.
- Controlled sources are replaced with their dual (e.g., VCVS are replaced with CCCS).

Similar considerations can be drawn for current inputs. The interested reader is referred to [32,33] for a more in-depth analysis of adjoint equivalent networks.

**Figure 6:**
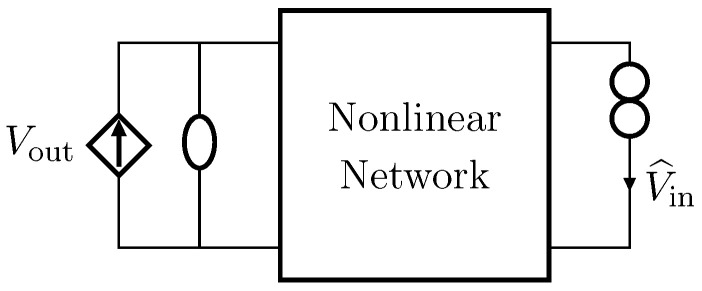
Adjoint network of the *Inverse System* shown in Figure 4c.

The adjoint transformation can be carried out either on the *Direct System* or directly on the *Inverse System*. For example, Figure 6 shows the adjoint network of the *Inverse System* of Figure 4c, where the nullator is replaced with a norator and vice versa, the input VCVS is substituted with a short circuit, while a CCCS is connected to the output port. The current flowing through the short circuit will be equal to ${\hat{V}}_{\mathrm{in}}$, whereas the input current will be equal to $V_{\mathrm{out}}$, i.e., the output voltage of the *Direct System* shown in Figure 4b. An equivalent inverse network can be obtained starting from the adjoint of the *Direct System* and applying then the rules presented in the previous subsections for deriving the *Inverse System*.

In the next section, we will present the inversion-based virtualization algorithm providing details on how to apply it for both the cases of actuator and sensor virtualization.

## 3. Direct–Inverse–Direct Chains

**Figure 7:**
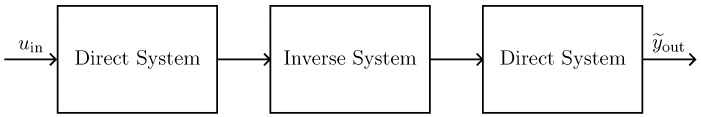
General Direct–Inverse–Direct Chain for the task of virtualization of audio transducers.

We now present a general block chain to perform virtualization of transducers. Such a chain, shown in Figure 7, is called *Direct–Inverse–Direct Chain* (DIDC) and was proposed in [17] for addressing the virtualization of loudspeakers. It is composed of three main blocks: two *Direct Systems*, and one *Inverse System*. The *Inverse System* is always implemented in the digital domain, whereas, according to the considered actuator or sensor application, only the first or the last *Direct System* is implemented in the digital domain since the other is the actual physical transducer. Moreover, in real scenarios, amplifiers could be present in-between blocks. Hence, gains should be considered at different stages of the processing chain for the algorithm to properly work.

The DIDC working principle is based on the assumption that the cascade of the *Inverse System* and the *Physical Direct System* is equivalent to the identity. This means that the digital processing chain allows us to somehow cancel out the behavior of the transducer such that the target behavior, i.e., the behavior of the digital *Direct System*, can be imposed. Hence, the proposed processing chain can be employed to accomplish the task of transducer virtualization (i.e., digitally altering the acoustic behavior of an audio transducer with the aim of mimicking the sound of a target transducer).

In the following two subsections, we will present application-specific DIDCs targeting both the cases of actuator and sensor virtualization.

### 3.1. Target-Inverse-Physical Chain (TIPC)

**Figure 8:**
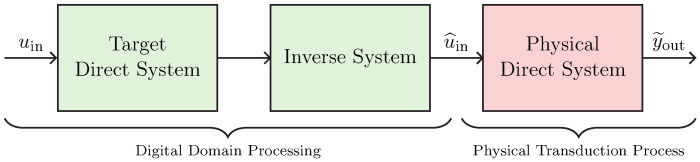
Target-Inverse-Physical Chain for the virtualization of audio actuators.

Let us consider the particular DIDC shown in Figure 8. Such a DIDC is specifically tailored for the task of actuator virtualization, and has been first proposed in [16] for deriving the loudspeaker virtualization algorithm. The green blocks are to be implemented in the digital domain, while the red block represents the actual physical transducer. In particular, the *Target Direct System* is the digital implementation of the actuator circuital model which we would like to obtain, whereas the *Inverse System* is the inverse circuital model of the *Physical Direct System*, which is the transducer itself. Hence, given that we are considering actuation, we may call this chain *Target-Inverse-Physical Chain* (TIPC), since the target behavior must be imposed in a pre-processing phase and thus before driving the actuator, i.e., the *Physical Direct System*.

For the case of loudspeaker virtualization, the input $u_{\mathrm{in}}$ is the electrical signal driving the loudspeaker, while the output signal ${\tilde{y}}_{\mathrm{out}}$ may be the output pressure or the velocity of the speaker diaphragm (usually represented in electrical equivalent models as a voltage signal and a current signal, respectively [1,16,17,27]). Then, in principle, the TIPC allows us to make a speaker A sound as a speaker B, where such a speaker B (i.e., the *Target Direct System*) can be a linearized or equalized version of A, or a different speaker. It follows that the more accurate the considered electrical models, the higher the performance of the algorithm. Nonetheless, in [17], it is shown that the algorithm is robust to parameter uncertainty, increasing the number of real scenarios in which it can be applied. In Section 5, we will show how to apply TIPC for the virtualization of a nonlinear compression driver, and how this can be accomplished in a simple and efficient fashion making use of Wave Digital Filters (WDFs).

It is worth adding that other variables aside currents and voltages could be taken into account to obtain the inverse of a given circuit. For example, for the case of loudspeakers, it might be convenient to consider the displacement of the diaphragm (i.e., the integral of the velocity of the diaphragm) as the output variable. In this case, another stage should be inserted into the processing chain for performing the integral of the velocity (which is a current variable in the electrical equivalent circuit) in the *Direct System*, and the derivative of the displacement in the *Inverse System*.

Finally, note that according to the type of virtualization, the blocks could be either linear or nonlinear. For example, all the three blocks could be nonlinear if the chain is exploited for imposing the nonlinear sonic behavior of a target transducer. Instead, if linearization is envisaged, one out of the three blocks will be linear (i.e., the *Target Direct System*). In this case, the purpose of virtualization is to improve the performance of the transducers by reducing the Total Harmonic Distortion (THD) imposing somehow the acoustic behavior of an ideal version of the transducer under consideration. Such a discussion is also valid for the specific DIDC that we introduce in the next subsection.

### 3.2. Physical-Inverse-Target Chain (PITC)

**Figure 9:**
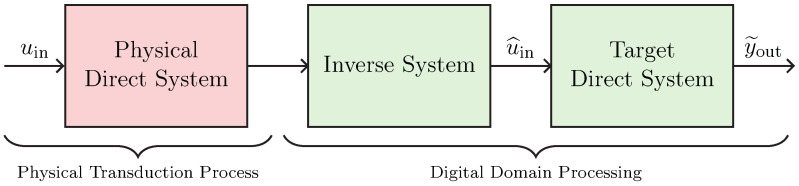
Physical-Inverse-Target Chain for the virtualization of audio sensors.

Let us now consider the DIDC shown in Figure 9, which is specifically designed to address the task of sensor virtualization. Such a DIDC can be considered as a flipped version of the TIPC, since the target behavior is imposed in a post-processing phase instead of a pre-processing phase. Once again, the green blocks are implemented in the digital domain, whereas the red block represents the actual physical transducer. The *Target Direct System* is the digital implementation of the sensor circuital model whose behavior we would like to impose, while the *Inverse System* is the inverse circuital model of the *Physical Direct System*, i.e., the sensor itself. Given that we are considering sensing, we call this version of the chain *Physical-Inverse-Target Chain* (PITC), since first the audio signal is acquired by means of the sensor, and then, after compensating for the physical behavior by means of the *Inverse System*, the signal is processed to impose the target acoustic response. For the case of microphone virtualization, the input signal $u_{\mathrm{in}}$ might be the acoustic pressure (i.e., a voltage) acquired by the sensor while the output signal is an electrical signal (e.g., a voltage) that is usually fed to an audio interface. In this scenario, therefore, the aim of the PITC is to modify the electrical signal as if it was acquired by another sensor, which can be a linearized or equalized version of the sensor under consideration or a different sensor. In Section 4, we will apply such a virtualization algorithm for altering the acoustic response of a condenser microphone.

Once again, input and output variable different from voltages and currents (e.g., displacement signals) can be considered by introducing integrators and derivators into the green blocks of the processing chain.

## 4. Sensor Virtualization: Application to Capacitive Microphones

**Figure 10:**
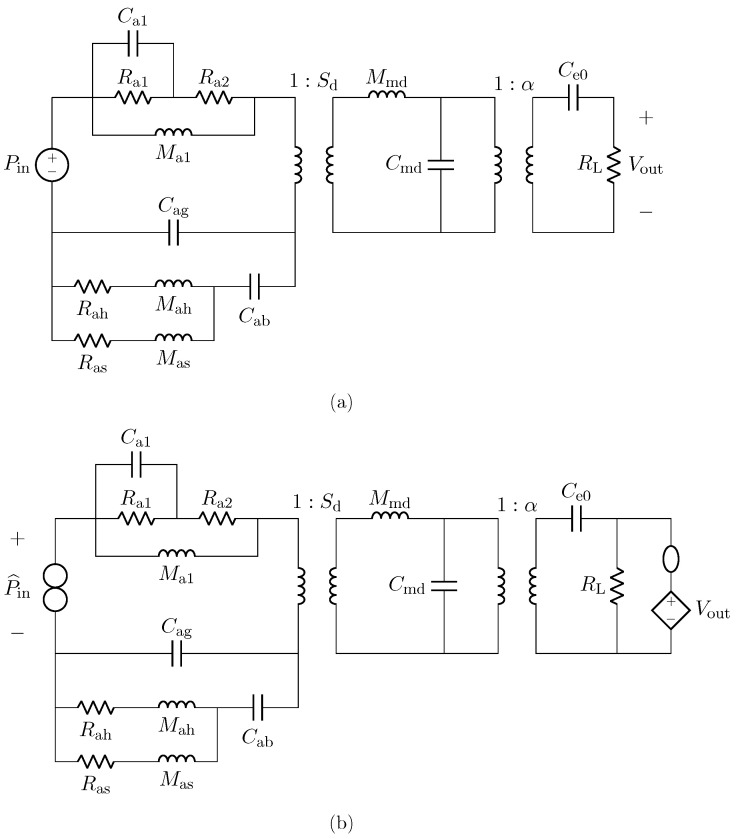
Circuital model of the capacitive microphone. (**a**) *Direct System*; (**b**) *Inverse System*.

In this section, we provide an example of sensor virtualization by taking into account a capacitive microphone as a case study. For this application, we employ a linear model, while an example of transducer virtualization based on nonlinear models will be provided in the next section. The microphone is described by means of the circuit shown in Figure 10a. The circuit is similar to the one presented in [1] and, it is composed of three subcircuits which represent, from left to right, the acoustic, mechanical, and electrical domains. In particular, $C_{\mathrm{ag}}$ is the acoustic compliance of the air gap, $R_{\mathrm{as}}$ and $M_{\mathrm{as}}$ model the acoustic resistance and mass of the back plate slots, $R_{\mathrm{ah}}$ and $M_{\mathrm{ah}}$ model the acoustic resistance and mass of the back plate holes, while the acoustic compliance of the back chamber is represented by $C_{\mathrm{ab}}$. Moreover, $R_{a1}$, $R_{a2}$, $M_{a1}$, and $C_{a1}$ model the free-field acoustic impedance. As far as the mechanical domain is concerned, $M_{\mathrm{md}}$ represents the mass of the diaphragm, whereas $C_{\mathrm{md}}$ is the mechanical compliance. Regarding the electrical domain, $C_{e0}$ is the electrical capacitance of the microphone, and $R_{L}$ models the input resistance of the Junction gate Field-Effect Transistor (JFET) to which the microphone capsule is usually connected.

The two transformers model the transduction between the physical domains, where $S_{d}$ is the diaphragm area and α is the electromechanical transduction factor. Finally, the input signal $P_{\mathrm{in}}$ is the acoustic pressure acquired by the microphone, while the output signal is the electrical voltage across resistor $R_{L}$. Table 2 reports the values of all the parameters for modeling both the Brüel & Kjær 4134 (hereafter referred to as BK4134) and the Brüel & Kjær 4146 (hereafter referred to as BK4146) electrostatic microphones [1,34,35].

**Figure 11:**
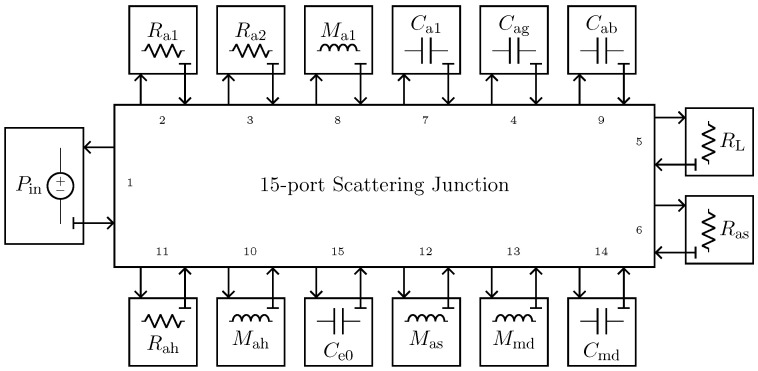
Possible WD implementation of the circuit shown in Figure 10a.

The implementation of the microphone circuital model in the digital domain can be carried out by employing different techniques [21,36,37]. In this work, we use Wave Digital Filters (WDFs) [21] since they proved to be suitable for an efficient implementation of both direct and inverse models of loudspeakers [16,17]. In particular, elements and topological interconnections can be realized as explained in [24], whereas nullors are encompassed into the scattering junction exploiting the double digraph decomposition of connection networks presented in [23]. Then, being the circuit linear, the Wave Digital (WD) structure can be solved with no iterative solvers by means of traditional techniques [21]. A possible WD realization of the circuit in Figure 10a is shown in Figure 11.

**Figure 12:**
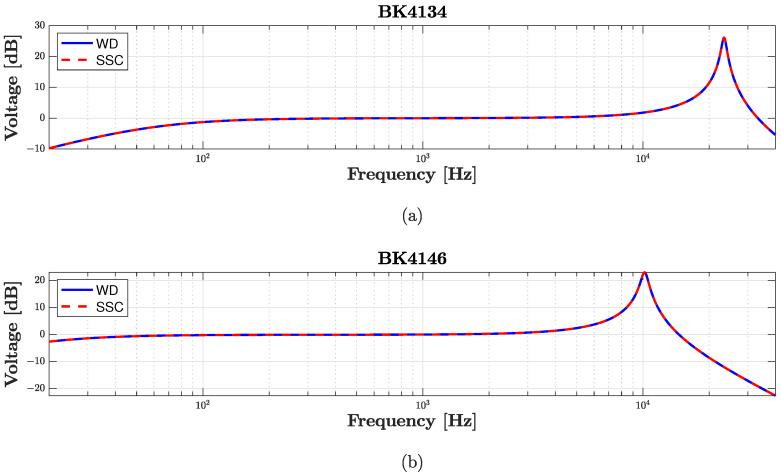
Comparison between the DFT of the impulse responses. The blue curves represent the WD implementation of the circuit in Figure 10a, while the red curves the Mathworks Simscape (SSC) implementation of the same circuit. (**a**) DFT of the impulse response for microphone BK4134; (**b**) DFT of the impulse response for microphone BK4146.

In order to test the accuracy of the WD implementation, we compare the Discrete Fourier Transform (DFT) of the impulse response obtained by simulating the reference circuit in the WD domain with the DFT of the impulse response obtained by simulating the same circuit in Mathworks Simscape (SSC), for both BK4134 and BK4146 microphones. The curves are then normalized with respect tot the pressure at 1 kHz as it is typically done to describe the microphone sensitivity. The results are shown in Figure 12 where the overlap between the continuous blue (WD) and the dashed red (SSC) curves confirms the accuracy of the representation. Looking at Figure 12b, we can appreciate that microphone BK4146 is characterized by a lower resonance frequency with respect to BK4134’s (see Figure 12b), which is due to a larger diaphragm mounted in the mic capsule.

### 4.1. Inverse Model Validation

**Figure 13:**
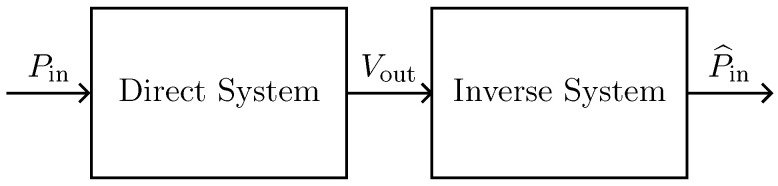
Processing chain for the validation of the *Inverse System*.

**Figure 14:**
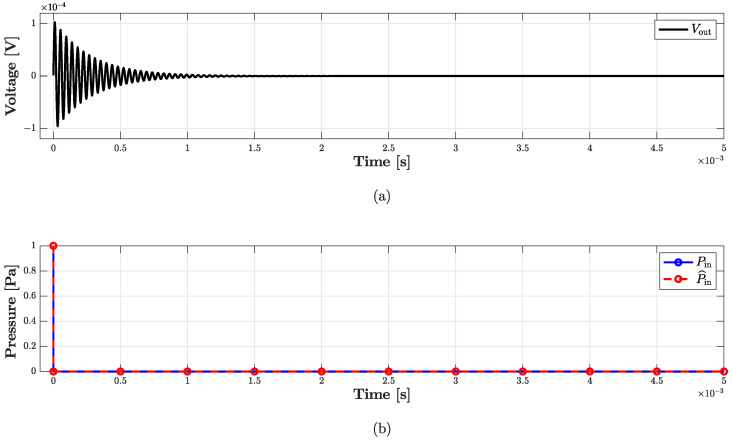
Validation of the *Inverse System* for microphone BK4134. (**a**) Output voltage of the *Direct System*; (**b**) comparison between $P_{\mathrm{in}}$ (dashed red curve) and ${\hat{P}}_{\mathrm{in}}$ (continuous blue curve) taking into account the processing chain shown in Figure 13.

In this subsection, we refer to the digital implementation of the microphone BK4134 equivalent circuit as *Direct System*. By applying the theorem presented in Section 2, it is possible to derive the *Inverse System* circuit shown in Figure 10b. In particular, since we are in a VIVO scenario, in order to design the *Inverse System*, once augmented the *Direct System* with a parallel connection of a nullator and a norator as explained in Section 2.2.1, we substitute the norator with a VCVS driven by $V_{\mathrm{out}}$ and the input source with the norator. The *Inverse System* can be implemented in the WD domain in a fully explicit fashion. In order to validate the *Inverse System* implementation, we consider the processing chain in Figure 13 which is composed of the cascade of the *Direct System* and the *Inverse System*, and we verify that the output of the cascade is equal to the input of the same cascade, i.e., ${\hat{P}}_{\mathrm{in}}=P_{\mathrm{in}}$. Figure 14 shows the results of such a test. We consider the input signal $P_{\mathrm{in}}$ of the *Direct System* to be an impulse and we compute the response of the microphone, which is shown in Figure 14a. Then, we feed the BK4134 *Inverse System* with the obtained voltage signal and we compare the output ${\hat{P}}_{\mathrm{in}}$ with the input $P_{\mathrm{in}}$. Looking at Figure 14b, we can notice that $P_{\mathrm{in}}$ and ${\hat{P}}_{\mathrm{in}}$ match perfectly since the output of the *Inverse System* is indeed an impulse. In order to further remark the accuracy of the *Inverse System* implementation, we compute the Root Mean Square Error (RMSE) between the input and output of the processing chain, obtaining a result below the machine precision. Finally, a similar test is carried considering the circuit equivalent parameters of microphone BK4146; even in this case, the RMSE is numerically zero.

### 4.2. Sensor Virtualization Test

**Figure 15:**
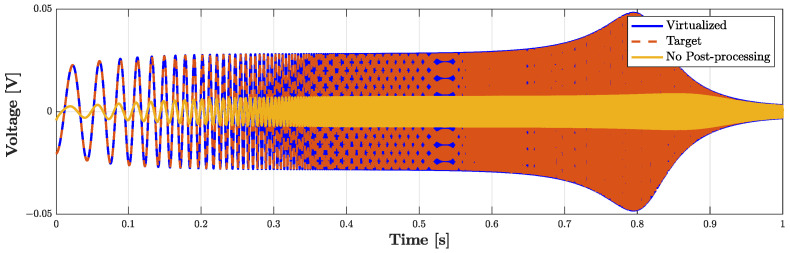
PITC-based virtualization algorithm. Output voltage signals of: the *Direct System*, i.e., BK4134, when no virtualization algorithm is present (“No Post-processing”), the *Target Direct System*, i.e., BK4146 (“Target”), and the PITC (“Virtualized”).

In this subsection, we provide an example of sensor virtualization. In particular, we employ the PITC-based algorithm presented in Section 3.2 for imposing the acoustic behavior of a target microphone. Let us suppose that the *Physical Direct System* is microphone BK4134 and that we would like to obtain a voltage signal $V_{\mathrm{out}}$ as if it was acquired by the microphone BK4146, i.e., the *Target Direct System*. It follows that the *Inverse System* is the circuital inverse model of microphone BK4134. The circuit parameters of both BK4134 and BK4146 are, once again, those listed in Table 2. Direct, inverse, and target systems are implemented in the WD domain as explained in the previous subsections. As input signal, we consider an exponential sine sweep defined as follows

(14) $$P_{\mathrm{in}}=\sin\left(2\pi f_{1}L\exp\left(\frac{k}{f_{s}L}\right)\right)\;,$$

where $f_{s}=96$ kHz is the sampling frequency, *k* the sample index, and $L=\frac{T}{\log\left(f_{2}/f_{1}\right)}$, with $f_{1}=20$ Hz as the starting frequency, $f_{2}=20$ kHz as the final frequency, and T=1 s as the total duration of the sweep. Figure 15 shows the result of such a test. The continuous yellow curve represents the output of the *Physical Direct System* when the PITC-based algorithm is not active, the dashed red curve represents the target behavior that we would like to obtain, while the continuous blue curve represents the output of the PITC, i.e., the output of the system when the algorithm is active. The overlap between blue and red curve is perfect given that the RMSE is below machine precision. The algorithm is thus able to impose the response of microphone BK4146 even if the pressure signal is acquired by means of BK4143, being characterized at the same time by real time capabilities. In fact, the algorithm implemented in a MATLAB script is able to process, on average, one sample in 1.12 µs, which is lower than $T_{s}=1/f_{s}=10.42$ µs. Note that, for these tests, the *Physical Direct System* is simulated by means of the WDF shown in Figure 11, but, in applications of interest, it represents the actual physical transducer. Moreover, the considered WDF is composed of just one topological junction to which all the elements are connected, but other solutions can be obtained. For example, 3-port topological adaptors can be employed when possible in order to create a WDF composed of multiple junctions and reduce the size of scattering matrices.

Finally, we would like to stress the fact that the circuital model shown in Figure 10a does not take into account the nonlinear behavior introduced by the JFET, which is typically connected to the microphone capsule, since it is not directly involved in the transduction process. It follows that, depending on the application, the electrical subcircuit might be modified by introducing the circuital elements downstream in order to accomplish a proper virtualization.

## 5. Actuator Virtualization: Application to Compression Drivers

**Figure 16:**
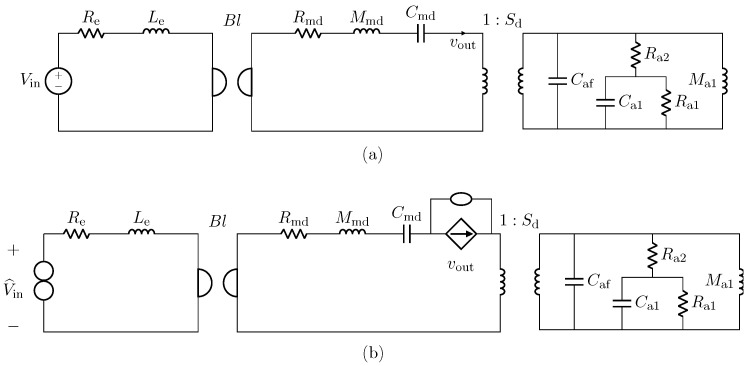
(**a**) Direct circuital model of the compression driver; (**b**) Inverse circuital model of the compression driver.

**Figure 17:**
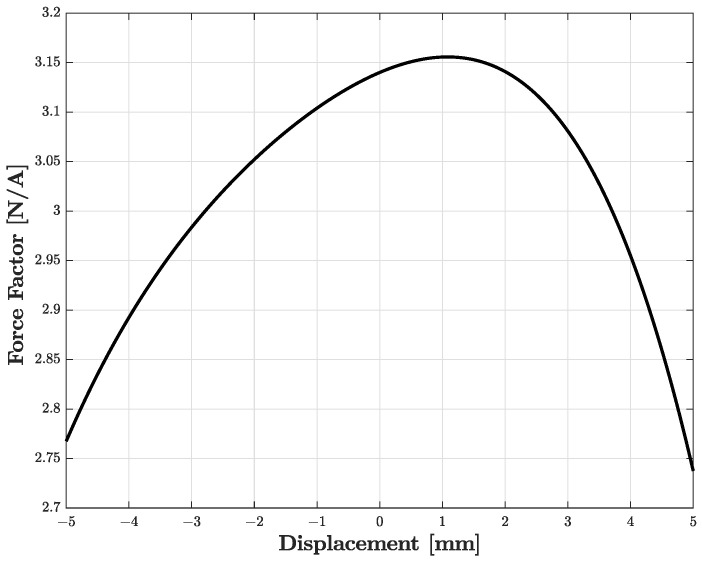
Nonlinear force factor Bl vs. displacement *x*.

We now consider a compression driver as an example of audio actuator. Such a transducer can be described by means of the circuit shown in Figure 16a adapted from [1], where we can anew distinguish three subsystems: the leftmost subcircuit modeling the electrical domain, the subcircuit in the middle modeling the mechanical domain, and the rightmost subcircuit modeling the acoustic domain. In particular, $L_{e}$ and $R_{e}$ are the electrical inductance and resistance of the voice coil, while $R_{\mathrm{md}}$, $M_{\mathrm{md}}$, and $C_{\mathrm{md}}$ are mechanical parameters: $R_{\mathrm{md}}$ models both the mechanical resistance and the resistance of the enclosure; $M_{\mathrm{md}}$ models the mass, also taking into account the voice coil, and $C_{\mathrm{md}}$ models both the compliance of the diaphragm and the compliance of the air in the enclosure. Moreover, $C_{\mathrm{af}}$ is the acoustic compliance of the front cavity. In addition, $R_{a1}$, $R_{a2}$, $M_{a1}$, and $C_{a1}$ model the free-field acoustic impedance at the driver throat. The gyrator represents the electromechanical transduction and is characterized by a nonlinear force factor Bl that can be modeled as follows [38]

(15) $$Bl\left(x\right)=Bl_{0}+Bl_{1}x+Bl_{2}x^{2}+Bl_{3}x^{3}+Bl_{4}x^{4}\;,$$

where *x* is the displacement of the diaphragm in millimeters obtained integrating velocity $v_{\mathrm{out}}$, and $Bl_{0},\ldots ,Bl_{4}$ are real polynomial coefficients. The ideal transformer, instead, models acoustic transduction as a function of the area of the diaphragm $S_{d}$. The input signal $V_{\mathrm{in}}$ is the electrical signal driving the loudspeaker, whereas as output signal we select the velocity $v_{\mathrm{out}}$, even though other signals can be chosen, e.g., the output pressure. Table 3 shows the values of the circuital parameters of the SEAS type 27TFF (H0831) compression driver model [1] (hereafter referred to as SEAS). Notably, the force factor coefficients are determined considering the typical Bl curve shown in Figure 17.

**Figure 18:**
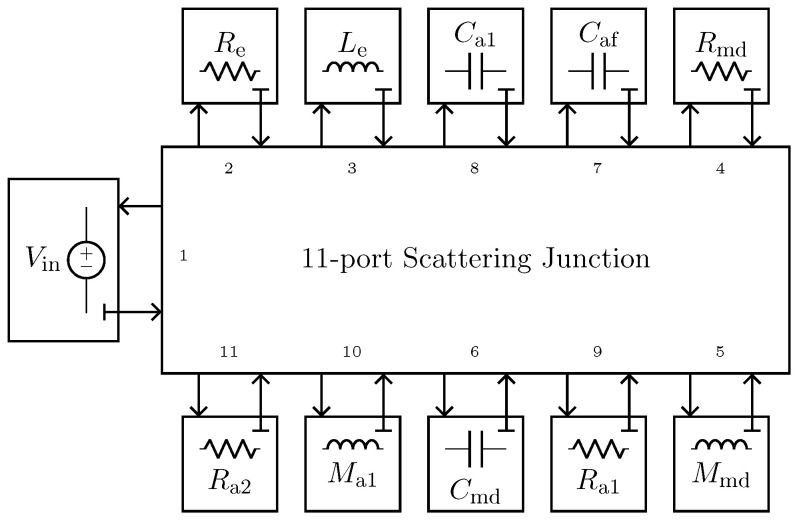
Possible WD implementation of the circuit shown in Figure 16a.

We implement the circuital model of the nonlinear compression driver using WDF principles. In particular, we encompass the gyrator into the scattering matrix by considering the method presented in [22], whereas the nonlinear force factor is modeled in the digital domain as explained in [16], leading to a fully explicit WDF structure that does not need iterative solvers to be implemented. The used WDF realization of the circuit in Figure 16a is shown in Figure 18.

The accuracy of the WDF modeling the *Direct System* has been validated by means of a comparison with Mathwork Simscape.

### 5.1. Inverse Model Validation

In this subsection, we validate the designed inverse of the loudspeaker circuital model shown in Figure 16a. We derive the *Inverse System* by applying the theorem presented in Section 2 and considering the VICO case. We do this by first augmenting the *Direct System* with a connection of a nullator and a norator in series to the same branch through which the output current flows as explained in Section 2.2.2; then, we substitute the norator with a CCCS driven by $v_{\mathrm{out}}$ and the input source with the norator. We finally implement the *Inverse System* in the WD domain in a fully explicit fashion, similarly to what done with the *Direct System*. In order to encompass both the nullor and the gyrator into the WD multi-port scattering junction, we employ again the method presented in [22].

**Figure 19:**
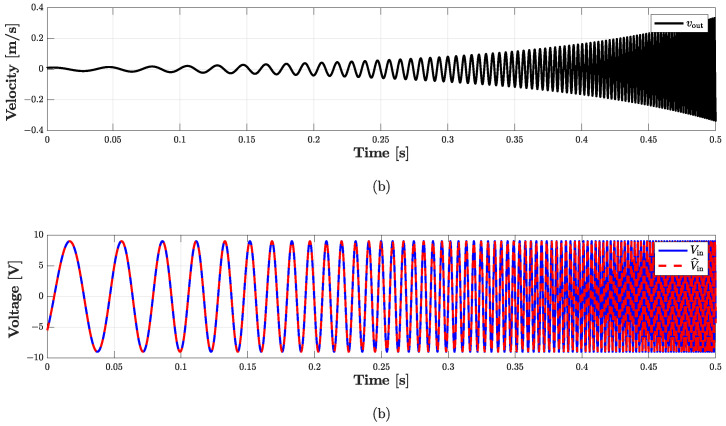
Validation of the *Inverse System* for loudspeaker SEAS. (**a**) Output voltage of the *Direct System*; (**b**) comparison between $V_{\mathrm{in}}$ (dashed red curve) and ${\hat{V}}_{\mathrm{in}}$ (continuous blue curve) taking into account a processing chain similar to that shown in Figure 13.

**Figure 20:**
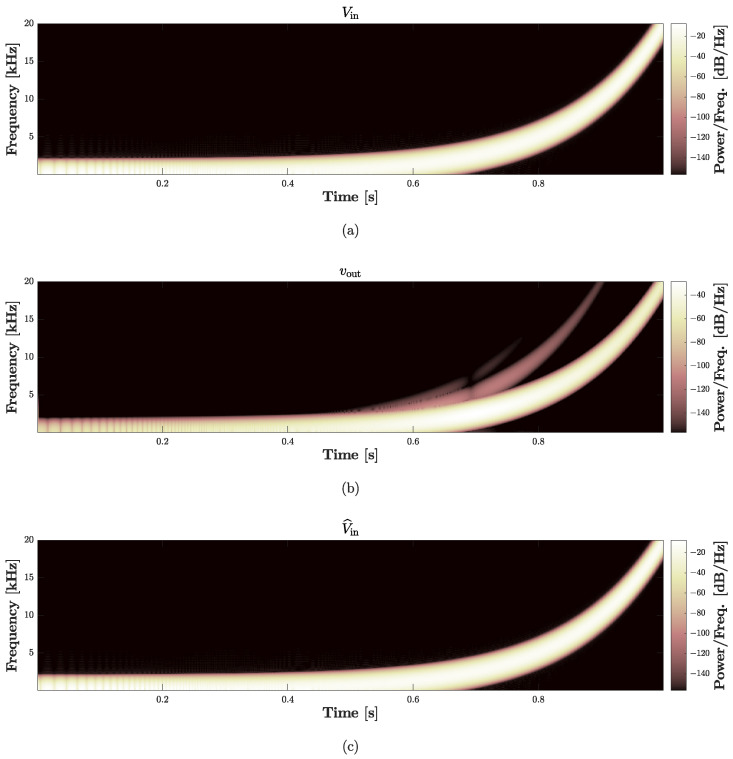
Validation of the *Inverse System*. (**a**) Input voltage $V_{\mathrm{in}}$ of the *Direct System*; (**a**) output velocity $v_{\mathrm{out}}$ (i.e., a current) of the *Direct System*; (**c**) output voltage ${\hat{V}}_{\mathrm{in}}$ of the *Inverse System*.

With the purpose of validating the WD implementation, we consider a processing chain similar to that shown in Figure 13, where now the *Direct System* is driven by voltage $V_{\mathrm{in}}$ and the *Inverse System* by the velocity signal $v_{\mathrm{out}}$, which, in turn, is the output current of the *Direct System*. If the implementation of the *Inverse System* is exact, we obtain ${\hat{V}}_{\mathrm{in}}=V_{\mathrm{in}}$. Figure 19 and Figure 20 show the results of such a test, the first in the time domain, while the second in the frequency domain. In particular, we consider as input signal of the cascade of *Direct System* and *Inverse System* the following exponential sine sweep

(16) $$V_{\mathrm{in}}=A\sin\left(2\pi f_{1}L\exp\left(\frac{k}{f_{s}L}\right)\right)\;,$$

where A=9 V is the amplitude, while all the other parameters are set as in Section 4.2. Figure 19 shows the signals obtained in the first 0.5 s of simulation: the dashed red curve represents the input of the *Direct System*, i.e., $V_{\mathrm{in}}$, and is perfectly overlapped with the continuous blue curve which represents the output of the *Inverse System*, i.e., ${\hat{V}}_{\mathrm{in}}$. To further analyze the performance of the *Inverse System*, in Figure 20 we also provide the spectrograms of the three signals involved in the validation chain. In particular, Figure 20a shows the spectrogram of $V_{\mathrm{in}}$, Figure 20b the spectrogram of $v_{\mathrm{out}}$, while Figure 20c the spectrogram of ${\hat{V}}_{\mathrm{in}}$. In the second plot, we can appreciate the nonlinear behavior of the loudspeaker circuital model since different harmonics appear over the frequency spectrum. Looking at the third plot, instead, we can further verify the action of the *Inverse System* since the harmonics characterizing $v_{\mathrm{out}}$ (i.e., the input of the *Inverse System*) nicely disappear, leading to a perfect match between $V_{\mathrm{in}}$ and ${\hat{V}}_{\mathrm{in}}$. Finally, both for time- and frequency-domain studies, we compute the RMSE between $V_{\mathrm{in}}$ and ${\hat{V}}_{\mathrm{in}}$ obtaining, once again, values below machine precision.

### 5.2. Actuator Linearization Test

**Figure 21:**
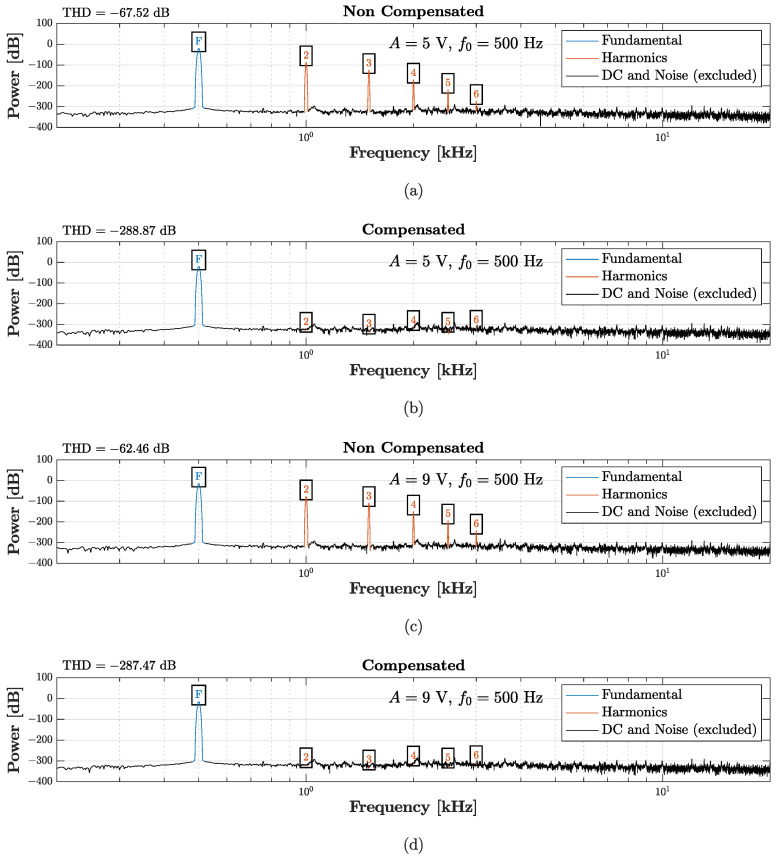
TIPC-based linearization algorithm. The first two plots are obtained considering A=5 V: (**a**) power spectrum of the *Physcal Direct System* output (“Non Compensated”); (**b**) power spectrum of the TIPC output (“Compensated”). Instead, the remaining rows are obtained considering A=9 V: (**c**) power spectrum of the *Physical Direct System* (“Non Compensated”); (**d**) power spectrum of the TIPC output (“Compensated”).

In a last application scenario, we show how the transducer linearization task can be accomplished as a particular case of the proposed virtualization algorithms. We aim, in fact, at eliminating the distortion effect introduced by the nonlinear behavior of the loudspeaker SEAS. In order to reach this goal, we employ the TIPC-based algorithm presented in Section 3.1 to impose the acoustic response of a target loudspeaker. In this application scenario, the *Target Direct System* is the linear version of the circuit shown in Figure 16a, which can be obtained by simply setting $Bl=Bl_{0}$ [16]. The parameters listed in Table 3 are again used for the WD implementations of the *Target Direct System*, the *Inverse System*, and the *Physical Direct System*. Contrary to what done in the microphone case, the desired behavior is imposed at the beginning of the processing chain since the physical transducer is an actuator. In order to test the chain, we set the input $V_{\mathrm{in}}=A\sin\left(2\pi f_{0}k/f_{s}\right)$, where *A* is the amplitude, *k* is the sample index, $f_{0}=500$ Hz is the fundamental frequency, and $f_{s}=96$ kHz is the sampling frequency. Moreover, in order to test the nonlinear system in different operating conditions, we consider two different amplitudes. Figure 21 shows the results of such a test. In particular, the figure shows the power spectra of the *Direct System* output (“Non Compensated”) and of the TIPC output (“Compensated”), together with the values of THD. Figure 21a,b are obtained by setting A=5 V, whereas Figure 21c,d are obtained by setting A=9 V. In both cases, the TIPC-based algorithm is able to suppress the harmonics introduced by the nonlinearity affecting the compression driver, while maintaining the content at the fundamental frequency $f_{0}$. This can be quantified looking at the THD reduction, which for both the tests is over 220 dB. As far as efficiency is concerned, instead, the algorithm, implemented in a MATLAB script, is able to run in real-time, processing on average one sample in 7.32 µs. Note that, even in this case, the *Physical Target System* is simulated but, in real scenarios, it represents the actual physical transducer.

The TIPC-based algorithm can be thus promising for improving the acoustic response of the loudspeaker on the fly by pre-processing the electrical signal driving the loudspeaker itself. Finally, it is worth stressing that the tested virtualization algorithm can be exploited not only to accomplish linearization but also to impose a desired nonlinear behavior, similarly to what already shown in [17].

## 6. Conclusions

In this paper, we described a general approach for the virtualization of audio transducers applicable to both sensors, like microphones, and actuators, like loudspeakers. We defined virtualization as the task of altering the acquired/reproduced signal by making it sound as if acquired/reproduced by another ideal or real audio sensor/actuator. In order to accomplish such a task, we started by reformulating Leuciuc’s theorem and proof for circuit inversion, providing case-specific guidelines on how to derive the inverse circuital model for all combinations of input and output variables. In particular, circuital inversion is achieved by augmenting the *Direct System* with a theoretical two-port called nullor, exploiting nullor equivalent models of short and open circuits [30]. We then presented two versions of the Direct–Inverse–Direct Chain which allow us to address virtualization for both sensors (Physical-Inverse-Target Chain) and actuators (Target-Inverse-Physical Chain). The chains are composed of three blocks: a *Physical Direct System*, which is the responsible for the actual transduction process, an *Inverse System*, which is the circuital inverse of the *Physical Direct System*, and a *Target Direct System*, which is the transducer characterized by the behavior that we would like to obtain. We exploited WDF principles to implement the digital blocks of such processing chains in a fully explicit fashion, i.e., without resorting to iterative solvers. Finally, we tested both the PITC-based and the TIPC-based algorithms for addressing microphone virtualization and linearization of a loudspeaker system with nonlinear compression driver.

Future work may concern first the extension of circuital inversion theory to the Multiple Input Multiple Output (MIMO) case, and then, by exploiting this new theory, the development of refined DIDC-based algorithms for addressing the case of array virtualization, both in sensing and actuation scenarios.

## Figures and Tables

**Table 1:** The four possible inversion scenarios.

Input Signal	Output Signal	Direct System	Inverse System
Voltage	Voltage	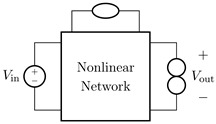	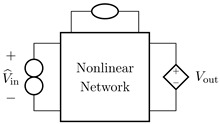
		**Network A.**	**Network B.**
Voltage	Current	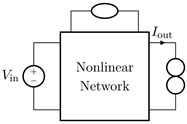	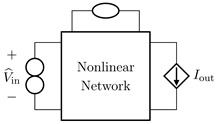
		**Network C.**	**Network D.**
Current	Voltage	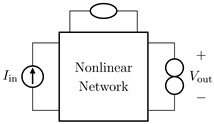	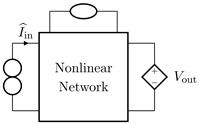
		**Network E.**	**Network F.**
Current	Current	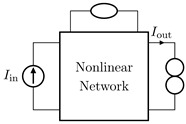	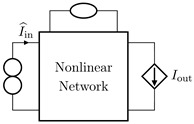
		**Network G.**	**Network H.**

**Table 2:** Parameters of the equivalent circuit for modeling BK4134 and BK4146 microphones.

Parameter	BK4134	BK4146	Parameter	BK4134	BK4146
$R_{a1}$ $\left[\frac{\mathrm{kg}}{m^{4}s}\right]$	$4.12\times 10^{6}$	$1.03\times 10^{6}$	$M_{\mathrm{ah}}$ $\left[\frac{\mathrm{kg}}{m^{4}s^{2}}\right]$	278.2	209.52
$R_{a2}$ $\left[\frac{\mathrm{kg}}{m^{4}s}\right]$	$6.54\times 10^{6}$	$1.66\times 10^{6}$	$C_{\mathrm{ab}}$ $\left[\frac{m^{4}s^{2}}{\mathrm{kg}}\right]$	$0.89\times 10^{-12}$	$4.76\times 10^{-12}$
$M_{a1}$ $\left[\frac{\mathrm{kg}}{m^{4}s^{2}}\right]$	54.83	27.44	$M_{\mathrm{md}}$ $\left[\mathrm{kg}\right]$	$3.69\times 10^{-6}$	$14.73\times 10^{-6}$
$C_{a1}$ $\left[\frac{m^{4}s^{2}}{\mathrm{kg}}\right]$	$1.95\times 10^{-12}$	$15.58\times 10^{-12}$	$C_{\mathrm{md}}$ $\left[\frac{m}{N}\right]$	$12.58\times 10^{-6}$	$26.55\times 10^{-6}$
$C_{\mathrm{ag}}$ $\left[\frac{m^{4}s^{2}}{\mathrm{kg}}\right]$	$9.12\times 10^{-15}$	$46.54\times 10^{-15}$	$C_{e0}$ $\left[F\right]$	$27.36\times 10^{-12}$	$90.72\times 10^{-12}$
$R_{\mathrm{as}}$ $\left[\frac{\mathrm{kg}}{m^{4}s}\right]$	$4.13\times 10^{3}$	444.58	$R_{L}$ $\left[Ω\right]$	$100\times 10^{6}$	$100\times 10^{6}$
$M_{\mathrm{as}}$ $\left[\frac{\mathrm{kg}}{m^{4}s^{2}}\right]$	18.8	6.24	$S_{d}$ $\left[m^{2}\right]$	$62.2\times 10^{-6}$	$248.3\times 10^{-6}$
$R_{\mathrm{ah}}$ $\left[\frac{\mathrm{kg}}{m^{4}s}\right]$	$99.93\times 10^{3}$	$86.45\times 10^{3}$	α $\left[\frac{N}{V}\right]$	121.17	140

**Table 3:** Parameters of the considered SEAS compression driver model.

Parameter	SEAS	Parameter	SEAS
$R_{a1}$ $\left[\frac{\mathrm{kg}}{m^{4}s}\right]$	$0.72\times 10^{6}$	$R_{e}$ $\left[Ω\right]$	4.9 Ω
$R_{a2}$ $\left[\frac{\mathrm{kg}}{m^{4}s}\right]$	$1.64\times 10^{6}$	$L_{e}$ $\left[H\right]$	$50\times 10^{-6}$
$M_{a1}$ $\left[\frac{\mathrm{kg}}{m^{4}s^{2}}\right]$	36.32	$Bl_{0}$ $\left[\frac{N}{A}\right]$	3.14
$C_{a1}$ $\left[\frac{m^{4}s^{2}}{\mathrm{kg}}\right]$	$30.11\times 10^{-12}$	$Bl_{1}$ $\left[\frac{N}{A\;\mathrm{mm}}\right]$	$2.7\times 10^{-2}$
$C_{\mathrm{af}}$ $\left[\frac{m^{4}s^{2}}{\mathrm{kg}}\right]$	$9.88\times 10^{-12}$	$Bl_{2}$ $\left[\frac{N}{A\;{\mathrm{mm}}^{2}}\right]$	$1\times 10^{-2}$
$R_{\mathrm{md}}$ $\left[\frac{\mathrm{kg}}{m}\right]$	0.92	$Bl_{3}$ $\left[\frac{N}{A\;{\mathrm{mm}}^{3}}\right]$	$1.2\times 10^{-3}$
$M_{\mathrm{md}}$ $\left[\mathrm{kg}\right]$	$298.64\times 10^{-6}$	$Bl_{4}$ $\left[\frac{N}{A\;{\mathrm{mm}}^{4}}\right]$	$2.2\times 10^{-4}$
$C_{\mathrm{md}}$ $\left[\frac{m}{N}\right]$	$14.1\times 10^{-6}$	$S_{d}$ $\left[m^{2}\right]$	$0.7\times 10^{-3}$

## Data Availability

Not applicable.
